# Microorganisms and Biochar Improve the Remediation Efficiency of *Paspalum vaginatum* and *Pennisetum alopecuroides* on Cadmium-Contaminated Soil

**DOI:** 10.3390/toxics11070582

**Published:** 2023-07-04

**Authors:** Jiahao Liang, Jiechao Chang, Jiayao Xie, Liquan Yang, Mohamed S. Sheteiwy, Abdel-Raouf A. Moustafa, Mohamed S. Zaghloul, Haiyan Ren

**Affiliations:** 1 College of Agro-Grassland Science, Nanjing Agricultural University, Nanjing 210095, China; 2 Department of Agronomy, Faculty of Agriculture, Mansoura University, Mansoura 35516, Egypt; 3 Botany Department, Faculty of Science, Suez Canal University, Ismailia 41522, Egypt

**Keywords:** phytoremediation, micro-remediation, biochar, cadmium pollution

## Abstract

Phytoremediation can help remediate potential toxic elements (PTE) in soil. Microorganisms and soil amendments are effective means to improve the efficiency of phytoremediation. This study selected three microorganisms that may promote phytoremediation, including bacteria (*Ceratobasidium*), fungi (*Pseudomonas mendocina*), and arbuscular-mycorrhizal fungi (AMF, *Funneliformis caledonium*). The effects of single or mixed inoculation of three microorganisms on the phytoremediation efficiency of *Paspalum vaginatum* and *Pennisetum alopecuroides* were tested under three different degrees of cadmium-contaminated soil (low 10 mg/kg, medium 50 mg/kg, and high 100 mg/kg). The results showed that single inoculation of AMF or *Pseudomonas mendocina* could significantly increase the biomass of two plants under three different degrees of cadmium-contaminated soil, and the growth-promoting effect of AMF was better than *Pseudomonas mendocina*. However, simultaneous inoculation of these two microorganisms did not show a better effect than the inoculation of one. Inoculation of *Ceratobasidium* reduced the biomass of the two plants under high concentrations of cadmium-contaminated soil. Among all treatments, the remediation ability of the two plants was the strongest when inoculated with AMF alone. On this basis, this study explored the effect of AMF combined with corn-straw-biochar on the phytoremediation efficiency of *Paspalum vaginatum* and *Pennisetum alopecuroides*. The results showed that biochar could affect plant biomass and Cd concentration in plants by reducing Cd concentration in soil. The combined use of biochar and AMF increased the biomass of *Paspalum vaginatum* by 8.9–48.6% and the biomass of *Pennisetum alopecuroides* by 8.04–32.92%. Compared with the single use of AMF or biochar, the combination of the two is better, which greatly improves the efficiency of phytoremediation.

## 1. Introduction

Potential toxic elements in soil are a global environmental problem with potential risks to human health [1]. PTE is naturally present in the soil. Due to the long-term evolution of the soil, this type of PTE has low concentration and stable properties [2]. Human activities, such as ore mining, industrial effluents and gas discharge, chemical fertilizer and pesticide use, and household waste, are the main sources of PTE in contaminated land [3]. These pollutants enter the soil through atmospheric deposition and irrigation, resulting in PTE pollution [4]. Most PTE is non-essential to plants, microorganisms, and the human body [5,6]. In addition to directly endangering plants and microorganisms living in the soil, PTE will eventually harm human health through the enrichment of the food chain [7,8]. Phytoremediation has been proven to be an effective means of controlling PTE [9]. It does not require excessive energy input and does not cause secondary pollution [10]. However, phytoremediation also has disadvantages, such as a long repair cycle, low economic efficiency, slow plant growth, and so on [11]. Therefore, improving the efficiency of phytoremediation is the main research direction [12].

Plants are closely related to soil microorganisms, and a small number of soil micro-organisms can promote phytoremediation through specific pathways [13]. Arbuscular mycorrhizal fungi (AMF) can form a symbiotic relationship with host plants, which is common in nature [14]. Numerous studies have shown that AMF can promote plant growth and improve the efficiency of phytoremediation [15,16]. Plant-growth-promoting rhizobacteria PGPR is another type of microorganism that can improve the efficiency of phytoremediation [17]. PGPR can produce substances that promote plant growth, such as auxin IAA, siderophores, etc. [18]. These substances can help plant growth and reduce the toxicity of PTE to plants. Screening and evaluating microorganisms are essential for improving phytoremediation efficiency [19].

In addition to microorganisms, soil amendments are also an effective method of improving phytoremediation efficiency [20]. Soil amendments can optimize the living environment of plants and microorganisms by improving the physical and chemical properties of soil, thereby improving the efficiency of phytoremediation [21]. Biochar is a highly suitable soil amendment for phytoremediation [22]. It is a solid carbonaceous material produced by the pyrolysis of animal and plant biomass at high temperatures [23]. Biochar is usually alkaline, which can increase the pH value of soil and reduce the toxicity of soil heavy metals to a certain extent [24]. Biochar can increase the cation-exchange capacity (CEC) of soil, thereby improving the soil’s adsorption capacity for heavy metals. Moreover, biochar contains many minerals, such as K, Ca, P, Na, Mg, etc., which contribute to plant growth [25]. In the field of phytoremediation, biochar is a soil amendment with great potential.

Phytoremediation faces the disadvantage of low economic benefits, and the use of conventional plants for phytoremediation will bring safety hazards [26]. *Paspalum vaginatum* is a warm-season perennial grass with rhizomes and stolons, and it possesses strong drought resistance, salt tolerance, and trampling tolerance, making it a popular turfgrass species [27]. Similarly, *Pennisetum alopecuroides* also has strong stress resistance. Moreover, *Pennisetum alopecuroides* can improve soil environments and enhance soil fertility. Therefore, in the process of building a lawn, *Pennisetum alopecuroides* is generally used as a pioneer grass species to improve the environmental conditions of the planting site. Using the method of building a lawn to remediate PTE in the soil can not only avoid the risk of PTE being transmitted through the food chain but also bring certain economic benefits. This study explored the effects of three microorganisms and biochar on two turfgrasses, which will provide theoretical support for the establishment of turf to repair soil heavy metal pollution.

## 2. Materials and Methods

### 2.1. Experimental Materials

#### 2.1.1. Initial Soil

The initial soil was taken from Nanjing Agricultural University Baima Base. The soil was taken from 0–5 cm farmland. After removing debris with a 5 mm sieve, the soil was sterilized by autoclave (121 °C, 120 min) [28]. The physical and chemical properties of the initial soil are shown in Table 1.

#### 2.1.2. Plant Material

In this experiment, *Paspalum vaginatum* and *Pennisetum alopecuroides* were selected as plant materials. The seeds of *Pennisetum alopecuroides* were hybrid seeds, and seeds of similar size were selected for the experiment. The seeds were purchased from Zhengzhou Kaiyuan Prataculture Technology Co., Ltd. (Zhengzhou, China). The *Paspalum vaginatum* lawn was obtained from Nanjing Agricultural University Baima Base, and fresh stem segments of the same size were collected for the experiment.

#### 2.1.3. Microorganism Materials

The tested microorganisms include bacteria (*Ceratobasidium*), fungi (*Pseudomonas mendocina*), and AMF (*Funneliformis caledonium*). Bacteria were cultured in an LB (Luria-Bertani) culture, and fungi were cultured in a PDB (Potato Dextrose Broth) (Shanghai Shengsi Biochemical Technology Co., Ltd., Shanghai, China) medium. AMF were cultured in sand culture. AMF spores purchased from the College of Resources and Environment of Nanjing Agricultural University were mixed with autoclaved sand, and maize seeds sterilized with NaClO solution were planted. AMF was propagated by two consecutive rounds of three-week culture.

#### 2.1.4. Biochar

The biochar used in this experiment was prepared from corn straw by high-temperature decomposition. It was purchased from Henan Lize Environmental Protection Technology Co., Ltd. (Henan, China). Pyrolysis temperature was 550 °C, and the time was 120 min.

### 2.2. Pot Experiment Design

The experiment was divided into two stages. In the first stage of the experiment, the effects of single or mixed inoculation of three microorganisms on the phytoremediation efficiency of *Paspalum vaginatum* and *Pennisetum alopecuroides* were tested under three different degrees of cadmium-contaminated soil (low 10 mg/kg, medium 50 mg/kg, and high 100 mg/kg). After the experiment, the microorganisms were evaluated by measuring and analyzing the biomass of plants and the cadmium concentration in plants. The optimal combination of microorganisms determined by the first stage test will continue to be used in the second stage test. In the second stage, different doses of biochar were added on the basis of the first stage to explore the effect of biochar and microorganisms on phytoremediation.

#### 2.2.1. The First Stage: Microorganism Evaluation Test

**Figure 1:**
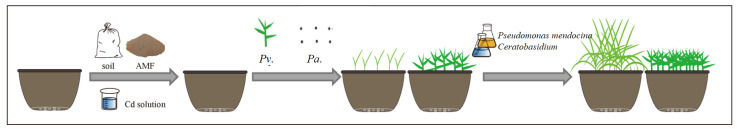
Experimental design of microorganism evaluation test. *Pv.* stands for *Paspalum vaginatum*, *Pa.* stands for *Pennisetum alopecuroides*.

The sterilized soil was mixed with CdCl_2_ · 2.5H_2_O solution to make three kinds of soil with different concentrations of cadmium (10 mg/kg, 50 mg/kg, and 100 mg/kg). Each pot was placed in 600 g soil. The contaminated soil was stabilized by placing it in the pot for a week. *Pennisetum alopecuroides* was planted in the form of seeds, and *Paspalum vaginatum* was planted through stem segments. Each pot eventually grew five plants. The three microorganisms were inoculated into the soil in a single or mixed form. The inoculation method of AMF was directly mixed with the original soil. After propagation with corn, the sand containing AMF was mixed with the original soil (Each pot was 5 g, and the control group was mixed with the same amount of sterilized sand). The inoculation method for bacteria and fungi was root injection. After the medium was shaken evenly, a 10 mL medium containing bacteria or fungi was directly injected into the rhizosphere soil at the seedling stage. The incubation time was 45 days; the greenhouse temperature was set at 27 degrees; the daily illumination time was 14 h (7 AM-9 PM); and the water was poured every two days. The experimental design of the microorganism evaluation test is shown in Figure 1.

#### 2.2.2. The Second Stage: Microorganism and Biochar Effect Test

**Figure 2:**
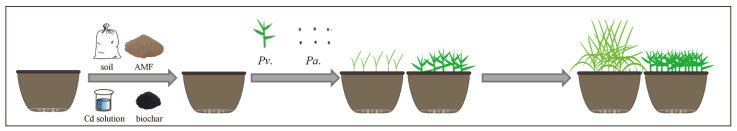
Experimental design of microorganism and biochar effect test. *Pv*. stands for *Paspalum vaginatum*, *Pa*. stands for *Pennisetum alopecuroides*.

Different doses of biochar were mixed with the original soil to prepare the soil with biochar content of 0%, 1%, 2%, and 3%. The researchers placed 600 g of soil into the pot and then added CdCl_2_ · 2.5H_2_O solution to make the cadmium content of the soil in the pot 50 mg/kg. The soil was placed in a pot for a week to make it stable. Two plants were planted and inoculated with AMF according to the first stage method. The plants were cultivated outside for two months and watered every two days. The experimental design of the microorganism and biochar effect test is shown in Figure 2.

### 2.3. Material Collection and Determination

At the end of the pot experiment, the aboveground parts of the plants were cut with scissors and placed in kraft paper bags in an oven (BIOBASE, DHG-9123A). The temperature was set to 105 °C for 30 min, with the aim of quickly stopping the metabolic reactions outside and avoiding the loss of dry matter [29]. Then, 30 min later, the temperature was lowered to 65 °C for 48 h. The plant samples were dried, and the dry weight of the aboveground parts of the plants was weighed using an electronic balance (Sartorius, SECURA324-1CN). After harvesting the aboveground parts, the pots were left for 1 week to reduce their water content. One week later, the soil in the pots was poured out, broken up, and the underground parts of the plants were picked up and placed in kraft paper bags. The dry weight of the underground parts was determined using the same method as the aboveground parts. PTE was determined by Inductively Coupled Plasma-Mass Spectrometry, and plant samples were digested by wet digestion. Then, 0.1 g of plant sample was placed in a 100 mL digestion tube, and after adding a small amount of water to wet the sample, 5 mL of sulfuric acid was added. After standing overnight, the digestion tube was placed in a digestion oven (YN-LWY84B) and digested at 180 °C for 15 min. After 15 min, the decoction tube was removed, and 2 mL of H_2_O_2_ was added. The tube was then shaken to complete the vigorous reaction, and digestion continued for 10 min to complete the reaction. The digested solution was diluted to 100 mL to keep the acid content within 5% to avoid damaging the instrument. An ICP-MS (Thermo Scientific iCAP Q ICP-MS) was used to determine and calculate the cadmium concentration in the plants.

### 2.4. Data Processing

Excel was used to record and save test data. IBM SPSS Statistics 19 was used to analyze the experimental data (Generalized Linear Models, Duncan analysis, *p* ≤ 0.05). Origin 2021 was used to draw the statistical graph of the results. The transport factors (TF) of the plants were calculated. Formula (1) is the calculation formula of TF.

(1) $$TF=\frac{Cd\;concentration\left(above-ground\;parts\right)}{Cd\;concentration\left(underground\;parts\right)}$$

## 3. Results

### 3.1. The First Stage: Microorganisms Evaluation Test Results

The variance analysis of plant biomass, plant cadmium content, and TF is shown in Table 2 (Supplementary Materials). Plants, microorganisms, and cadmium all have a significant effect on the measured indicators.

#### 3.1.1. Biomass of *Paspalum vaginatum* and *Pennisetum alopecuroides*

**Figure 3:**
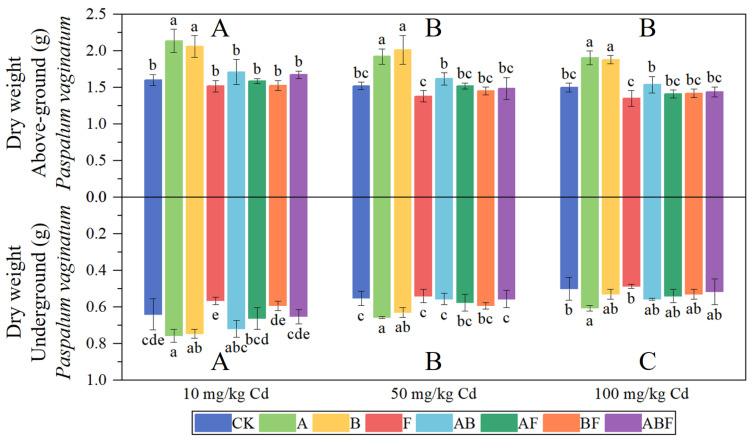
The biomass of *Paspalum vaginatum* under different microorganisms and cadmium concentrations. (CK represents no processing, A represents AMF, B represents bacteria, F represents fungi, AB represents AMF + bacteria, AF represents AMF + fungi, BF represents bacteria + fungi, and ABF represents AMF + bacteria + fungi. Duncan’s multiple-range test was used, *n* = 3, *p* ≤ 0.05, Different lowercase letters indicate significant differences in plant biomass between different microorganism treatment groups at the same cadmium level. A, B, C indicate the difference between different cadmium concentration treatment groups).

Figure 3 shows the biomass of *Paspalum vaginatum*. A single inoculation of AMF or bacteria significantly increased the biomass of *Paspalum vaginatum*. The effect of mixed inoculation of AMF and bacteria on the biomass of *Paspalum vaginatum* was less than that of the single inoculation of AMF or bacteria. The single inoculation of AMF increased the aboveground dry weight of *Paspalum vaginatum* by 26.48–33.47% and the underground dry weight by 18.22–21.33%. A single inoculation of bacteria increased the aboveground biomass of *Paspalum vaginatum* by 22.56–32.38% and the underground biomass by 6–16.67%. Fungi did not show a growth-promoting effect on *Paspalum vaginatum.*

**Figure 4:**
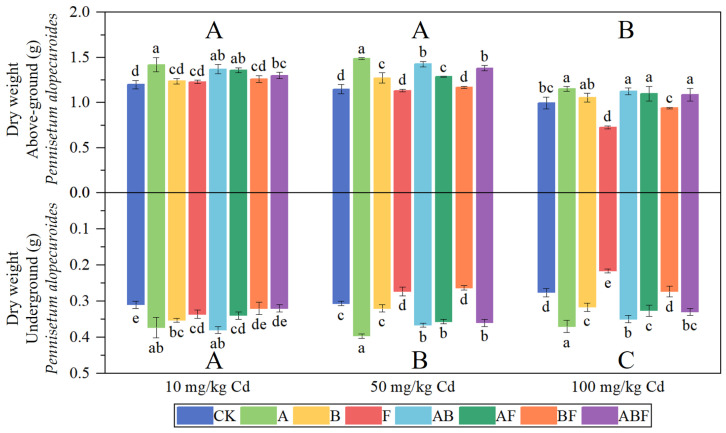
The biomass of *Pennisetum alopecuroides* under different microorganisms and cadmium concentrations. (CK represents no processing, A represents AMF, B represents bacteria, F represents fungi, AB represents AMF + bacteria, AF represents AMF + fungi, BF represents bacteria + fungi, and ABF represents AMF + bacteria + fungi. Duncan’s multiple-range test was used, *n* = 3, *p* ≤ 0.05, Different lowercase letters indicate significant differences in plant biomass between different microorganism treatment groups at the same cadmium level. A, B, C indicate the difference between different cadmium concentration treatment groups).

Figure 4 shows the biomass of *Pennisetum alopecuroides*. The single inoculation of AMF has significantly increased the plant’s biomass. The single inoculation of AMF increased the aboveground dry weight of *Pennisetum alopecuroides* by 15.72–29.28% and the underground biomass by 20.43–33.73%. Mixed inoculation of bacteria and AMF also significantly increased the biomass of *Pennisetum alopecuroides*. A single inoculation of bacteria significantly increased the underground biomass of *Pennisetum alopecuroides*. When the soil cadmium concentration was high, the single inoculation of fungi reduced the biomass of *Pennisetum alopecuroides*. Overall, among all treatment groups, the single inoculation of AMF had the highest growth effect.

#### 3.1.2. Cadmium Concentration in Plants

**Figure 5:**
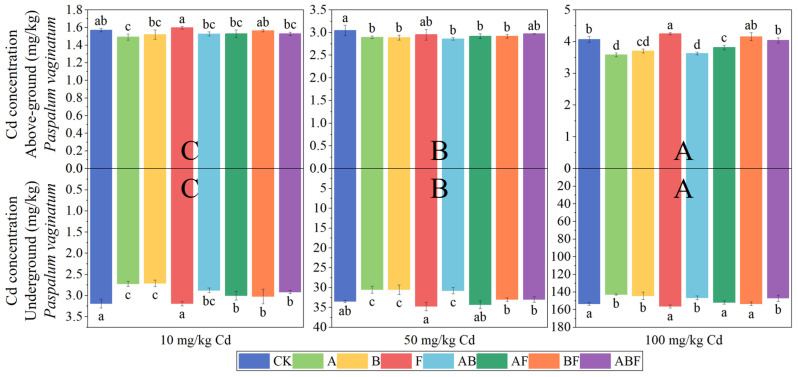
Cadmium concentration in *Paspalum vaginatum* under different microorganisms and cadmium concentrations. (CK represents no processing, A represents AMF, B represents bacteria, F represents fungi, AB represents AMF + bacteria, AF represents AMF + fungi, BF represents bacteria + fungi, and ABF represents AMF + bacteria + fungi. Duncan’s multiple-range test was used, *n* = 3, *p* ≤ 0.05, Different lowercase letters indicate significant differences in cadmium concentration in plants from different microorganisms treatment groups at the same cadmium level. A, B, C indicate the difference between different cadmium concentration treatment groups).

Figure 5 shows the cadmium concentration in *Paspalum vaginatum*. A single inoculation of AMF significantly reduced the cadmium concentration in *Paspalum vaginatum*. The aboveground cadmium concentration was reduced by 5.03–12.03%, and the underground cadmium concentration was reduced by 6.79–14.64%.

**Figure 6:**
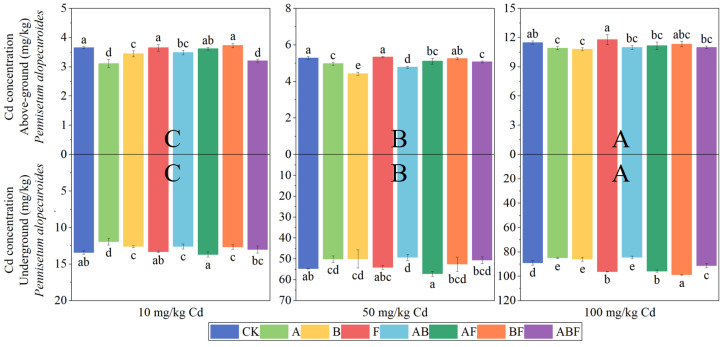
Cadmium concentration in *Pennisetum alopecuroides* under different microorganisms and cadmium concentrations. (CK represents no processing, A represents AMF, B represents bacteria, F represents fungi, AB represents AMF + bacteria, AF represents AMF + fungi, BF represents bacteria + fungi, and ABF represents AMF + bacteria + fungi. Duncan’s multiple-range test was used, *n* = 3, *p* ≤ 0.05, Different lowercase letters indicate significant differences in cadmium concentration in plants from different microorganism treatment groups at the same cadmium level. A, B, C indicate the difference between different cadmium concentration treatment groups).

Figure 6 shows the cadmium concentration in *Pennisetum alopecuroides*. A single inoculation of AMF or bacteria significantly reduced the cadmium concentration in *Pennisetum alopecuroides*. A single inoculation of fungi significantly increased the cadmium concentration in the underground part of *Pennisetum alopecuroides*. When the cadmium treatment concentration was 100 mg/kg, the inoculation of fungi significantly increased the underground cadmium concentration of *Pennisetum alopecuroides*.

#### 3.1.3. Transport Factors of Cadmium

**Figure 7:**
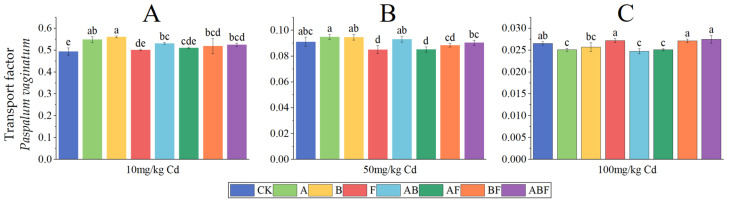
Transport factors of *Paspalum vaginatum* under different microorganisms and cadmium concentrations. (CK represents no processing, A represents AMF, B represents bacteria, F represents fungi, AB represents AMF + bacteria, AF represents AMF + fungi, BF represents bacteria + fungi, and ABF represents AMF + bacteria + fungi. Duncan’s multiple-range test was used, *n* = 3, *p* ≤ 0.05, Different lowercase letters indicate significant differences in the cadmium transport factors of plants from different microorganism treatment groups under the same cadmium level. A, B, C indicate the difference between different cadmium concentration treatment groups).

The cadmium transport factors of *Paspalum vaginatum* are shown in Figure 7. As the concentration of cadmium treatment increased, the cadmium transport factors of *Paspalum vaginatum* decreased. At a soil cadmium concentration of 10 mg/kg, the cadmium transport factors of the microorganism-treated group were higher than that of the control group, and bacteria-treated *Paspalum vaginatum* had the highest transport factors. At a soil cadmium concentration of 50 mg/kg, the cadmium transport factors of the fungus-treated group and the AMF + fungus-treated group were significantly lower than that of the control group, while there was no significant difference between the other groups and the control group. At a soil cadmium concentration of 100 mg/kg, the cadmium transport factors of the AMF, AMF + bacteria, and AMF + fungus treatment groups were significantly lower than that of the control group.

**Figure 8:**
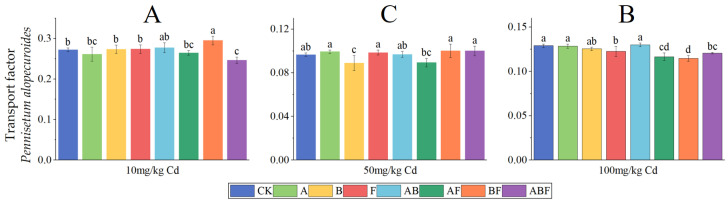
Transport factors of *Pennisetum alopecuroides* under different microorganisms and cadmium concentrations. (CK represents no processing, A represents AMF, B represents bacteria, F represents fungi, AB represents AMF + bacteria, AF represents AMF + fungi, BF represents bacteria + fungi, and ABF represents AMF + bacteria + fungi. Duncan’s multiple-range test was used, *n* = 3, *p* ≤ 0.05, Different lowercase letters indicate significant differences in the cadmium transport factors of plants from different microorganism treatment groups under the same cadmium level. A, B, C indicate the difference between different cadmium concentration treatment groups).

The cadmium transport factors of *Pennisetum alopecuroides* are shown in Figure 8. As the concentration of cadmium treatment increased, the cadmium transport factors of *Pennisetum alopecuroides* showed a trend of first decreasing and then increasing. When the soil cadmium concentration was 10 mg/kg, the cadmium transport factors of the bacterial treatment group were significantly lower than those of the control group. At a soil cadmium concentration of 50 mg/kg, the cadmium transport factors of the fungi and AMF + bacteria treatment groups were significantly lower than those of the control group. At a soil cadmium concentration of 100 mg/kg, the cadmium transport factors of the fungi, AMF + fungi, and AMF + bacteria + fungi treatment groups were significantly lower than those of the control group.

### 3.2. The Second Stage: Microorganism and Biochar Effect Test Results

The variance analysis of plant biomass, plant cadmium content, and TF is shown in Table 3 (Supplementary Materials). Biochar had a significant effect on the measured indicators.

#### 3.2.1. Biomass of *Paspalum vaginatum* and *Pennisetum alopecuroides*

**Figure 9:**
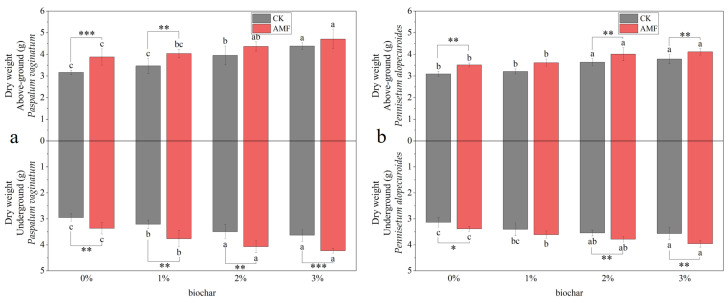
The biomass of *Paspalum vaginatum* (**a**) and *Pennisetum alopecuroides* (**b**) under different concentrations of biochar and AMF treatment. (Duncan multiple-range test was used, *n* = 6, *p* ≤ 0.05, Different lowercase letters indicate significant differences in plant biomass in different concentrations of biochar treatment groups under the same microorganisms treatment. The asterisk represents the significant difference between the AMF addition group and the control group under the same biochar concentration treatment, and a one-way analysis of variance is used. CK represents no processing. * *p* < 0.05, ** *p* < 0.01, *** *p* < 0.001).

Figure 9a shows the biomass of *Paspalum vaginatum* under different concentrations of biochar and AMF treatments. The biomass of *Paspalum vaginatum* increased with the increase in biochar application concentration. When the biochar application rates were 0% and 1%, the AMF inoculation significantly increased the aboveground biomass of the plant. The inoculation of AMF significantly increased the underground biomass of *Paspalum vaginatum*. Compared to the control group, the biomass of *Paspalum vaginatum* increased by 7.34–22.48% in the group treated with AMF.

Figure 9b presents the biomass of *Pennisetum alopecuroides* under different concentrations of biochar and AMF treatment. As the concentration of applied biochar increased, the biomass of *Pennisetum alopecuroides* exhibited an upward trend. In treatments with biochar concentrations of 0%, 2%, and 3%, inoculation of AMF significantly increased plant biomass. Compared with the control group without AMF inoculation, the biomass of *Pennisetum alopecuroides* in the AMF treatment group increased by 6.11–13.42%.

#### 3.2.2. Cadmium Concentration in Plants

**Figure 10:**
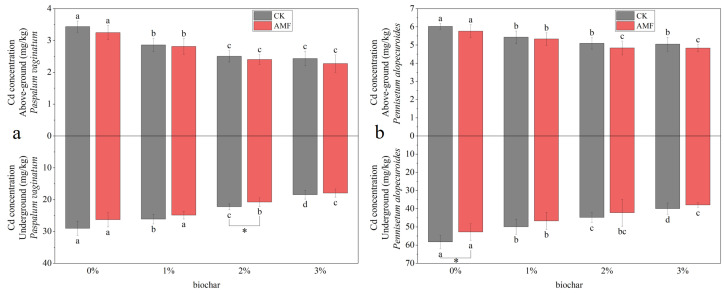
Cadmium concentration in *Paspalum vaginatum* (**a**) and *Pennisetum alopecuroides* (**b**) under different concentrations of biochar and AMF treatment. (Duncan multiple-range test was used, *n* = 6, *p* ≤ 0.05. Different lowercase letters indicate significant differences in cadmium concentration in plants treated with different concentrations of biochar under the same microorganism treatment. The asterisk represents the significant difference between the AMF addition group and the control group under the same biochar concentration treatment, and a one-way analysis of variance is used. CK represents no processing).

Figure 10a shows the cadmium concentrations in *Paspalum vaginatum* under different concentrations of biochar and AMF treatments. As the application concentration of biochar increased, the cadmium concentration in *Paspalum vaginatum* decreased gradually. The use of 3% of biochar reduced the aboveground cadmium concentration by 29.21% and the underground concentration by 36.37%. Compared with the control group, AMF treatment decreased the cadmium concentration accumulated in *Paspalum vaginatum* by 1.57% to 9.29%.

Figure 10b shows the cadmium concentrations in *Pennisetum alopecuroides* under different concentrations of biochar and AMF treatments. As the concentration of biochar increased, the cadmium concentration in *Pennisetum alopecuroides* declined gradually. The use of 3% of biochar reduced the aboveground cadmium concentration by 16.24% and the underground concentration by 31.35% compared with the control group; AMF treatment reduced the cadmium concentration accumulated in *Pennisetum alopecuroides* by 1.84–9.42%.

#### 3.2.3. Transport Factors of Cadmium

**Figure 11:**
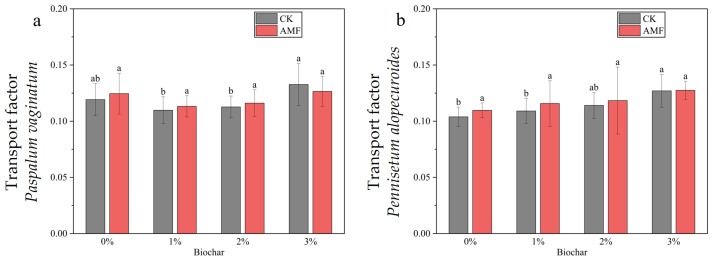
Transport factors of *Paspalum vaginatum* (**a**) and *Pennisetum alopecuroides* (**b**) under different concentrations of bio-char and AMF treatment. (Duncan multiple-range test was used, *n* = 6, *p* ≤ 0.05; different lowercase letters indicate significant differences in cadmium transport factors in plants treated with different concentrations of biochar under the same microorganism treatment. CK represents no processing).

Figure 11a shows the transport factors of *Paspalum vaginatum* under different concentrations of biochar and AMF treatments for cadmium. As the concentration of biochar applied increased, the cadmium transport factors of *Paspalum vaginatum* first decreased and then increased.

Figure 11b shows the transport factors of cadmium in the *Pennisetum alopecuroides* under the treatment of different concentrations of biochar and AMF. As the application concentration of biochar increased, the transport factors of cadmium in the *Pennisetum alopecuroides* continued to increase.

## 4. Discussion

### 4.1. Growth-Promoting Effect of Microorganisms

The *Pseudomonas mendocina* and *Ceratobasidium* used in this experiment were collected from the Qixia Mountain Pb-Zn mine area in Nanjing. Our previous studies have shown that these microorganisms have high production of indole acetic acid (IAA) and phosphorus solubility. IAA regulates plant growth, and studies have shown that microorganisms that produce IAA and have phosphorus solubility can increase plant biomass and heavy metal accumulation [30,31,32]. The symbiotic relationship between AMF and host plants increased the utilization efficiency of soil nutrients by host plants. Therefore, the tested microorganisms have the potential to improve the efficiency of phytoremediation.

Previous studies have reported that *Pseudomonas mendocina* can degrade PBAT [33]. The previous study also found that *Pseudomonas mendocina* has excellent denitrification ability and can help purify surface water pollution [34]. In this study, we found that inoculation with *Pseudomonas mendocina* could increase the biomass of *Paspalum vaginatum* and *Pennisetum alopecuroides* under cadmium-contaminated soil, but did not show a better enhancement effect when mixed with AMF.

It has been reported previously that *Ceratobasidium* stimulates the germination of orchid seeds and the development of seedlings [35]. The previous study also found that *Ceratobasidium* is a pathogen through gene sequence comparisons [36]. In this experiment, we found that *Ceratobasidium* inhibited plant growth and confirmed that *Ceratobasidium* is a pathogenic bacterium, but its ability to stimulate seed germination indicates that its metabolites may contain substances that facilitate seed germination. Although *Ceratobasidium* could produce IAA and phosphorus solubility, no promoting effect on plant growth was observed in this experiment.

The inoculation of AMF at four concentrations of cadmium increased the biomass of *Paspalum vaginatum* and *Pennisetum alopecuroides* to varying degrees. It has been reported that AMF can form a symbiotic relationship with plants, extend the roots of plants, increase the soil volume available to plants, continuously provide mineral nutrients to host plants, enhance the water absorption capacity of plant roots, and reduce pathogen damage to roots [37]. Previous studies found that AMF can promote plant uptake of heavy metals at low soil heavy metal concentrations, and at higher levels of soil heavy metal concentrations, AMF can bind with heavy metals to reduce their bioavailability and alleviate plant damage caused by heavy metals, increasing plant biomass and heavy metal tolerance while reducing heavy metal concentration in plants [16]. The application of AMF can slightly reduce the cadmium concentration in the plant body and significantly increase the biomass of plants. Research has found that AMF can mitigate the adverse effects of environmental factors by enhancing nutrient uptake, maintaining ion balance, improving antioxidant systems, and protecting enzyme activity [38].

### 4.2. Effect of Biochar on Phytoremediation

After applying biochar, there were significant changes in the biomass of both *Paspalum vaginatum* and *Pennisetum alopecuroides*. The unique chemical properties of biochar help to reduce the bioavailability of heavy metals in polluted soil. In addition, biochar can help to improve the soil environment, such as permeability, thus enhancing crop productivity [39]. Previous studies have found that peanut-shell-biochar significantly reduced the bioavailable concentration of Cr, Ni, As, Cd, and Pb in the soil as well as their accumulation in Brassica rapa [40]. The raw materials, pyrolysis temperature, and duration of biochar will affect its adsorption efficiency [41]. Generally, the stability and pH of biochar pyrolyzed at high temperatures are higher, and the effect is better [42]. The physical adsorption capacity of biochar is affected by van der Waals force. The size of the van der Waals force depends on its pore size and specific surface area, which is closely related to the preparation of raw materials [43]. Biochar has chemical adsorption characteristics, and the ions on the surface of biochar can exchange with PTE to stabilize PTE in soil [44]. Usually, the addition of biochar can reduce the effectiveness of PTE and reduce the concentration of PTE in plants [45]. However, some studies have shown that the chemical properties of biochar may enhance the absorption of PTE by plants [46]. This may be due to the fact that the absorption of PTE by plants is affected by the state of PTE in soil. The addition of biochar will change the state of PTE in soil. This change is mainly affected by the amount of PTE applied and the chemical composition contained. PTE changes not only soil pH but also soil physical properties. The change may also affect the effectiveness of PTE. Therefore, some biochar can increase the effectiveness of soil PTE, thereby enhancing the absorption of PTE by plants [47]. In summary, biochar is a highly suitable material for plant restoration, and using AMF based on biochar application can achieve better results.

### 4.3. Remediation Ability of Paspalum vaginatum and Pennisetum alopecuroides

In this study, with the increase of cadmium concentration treatment, the biomass of both *Paspalum vaginatum* and *Pennisetum alopecuroides* decreased, while the decrease in *Paspalum vaginatum* was smaller than that in *Pennisetum alopecuroides*, and *Paspalum vaginatum* accumulated more biomass during the same period. It was previously reported that the biomass of *Paspalum vaginatum* varied slightly in different degrees of high salinity combined with pollution sludge [48]. The high stability of *Paspalum vaginatum* in a contaminated soil environment is a major advantage as a phytoremediation material.

In this study, *Paspalum vaginatum* exhibited strong resistance. *Pennisetum alopecuroides* was insensitive to low to moderate cadmium concentrations. The main accumulation sites for both plants for cadmium were roots. Previous studies have screened a variety of cadmium-tolerant genes from *Paspalum vaginatum* and speculated that these genes may be involved in metal chelation, sugar metabolism, and other mechanisms [49].

When the soil cadmium concentration was low, the transport factors of cadmium in *Paspalum vaginatum* were greater than that in *Pennisetum alopecuroides*. However, when the soil cadmium concentration rose to 100 mg/kg, the transport factors of cadmium in *Paspalum vaginatum* were much less than that in *Pennisetum alopecuroides*. *Paspalum vaginatum* avoided damage to the aboveground part by controlling the transport of cadmium to the aboveground part, which gave it a higher tolerance to cadmium. Therefore, in a highly cadmium-polluted soil environment, *Paspalum vaginatum* can exert better remediation effects, while in a low cadmium-polluted environment, *Pennisetum alopecuroides* can accumulate more cadmium.

## 5. Conclusions

The two plants selected in this study have good remediation potential. Among them, *Pennisetum alopecuroides* has a higher accumulation ability in low-concentration cadmium-contaminated soil, while *Paspalum vaginatum* is more suitable as a plant material under high cadmium pollution. Single or mixed inoculation of AMF and bacteria can increase the biomass of the two plants, and the performance of the single inoculation of AMF is the best. The use of corn straw biochar reduced the absorption efficiency of cadmium by the two plants, alleviated the pressure of cadmium on plants, and increased the biomass of plants.

## Figures and Tables

**Table 1:** Physicochemical properties of initial soil.

Cd Concentration	pH	Electric Conductivity	Ammoniacal Nitrogen	Nitrate Nitrogen	Available Phosphorous
0.31 ± 0.06 (mg/kg)	6.51 ± 0.17	65.26 ± 9.14 (μS/cm)	14.37 ± 1.36 (mg/kg)	11.33 ± 1.23 (mg/kg)	17.58 ± 2.36 (mg/kg)

**Table 2:** The first stage variance analysis table.

Index	Plant	Microorganism	Cadmium	Plant × Microorganism	Plant × Cadmium	Cadmium × Microorganism	Plant × Microorganism × Cadmium
Dry weight (aboveground)	1105.098 <0.001 ***	66.946 <0.001 ***	104.935 <0.001 ***	26.749 <0.001 ***	21.024 <0.001 ***	0.947 0.513	1.635 0.083
Dry weight (underground)	2533.946 <0.001 ***	27.317 <0.001 ***	83.807 <0.001 ***	2.618 <0.05 *	31.521 <0.001 ***	1.221 0.273	3.205 <0.001 ***
Cd Plant (aboveground)	29,275.762 <0.001 ***	31.308 <0.001 ***	18,345.585 <0.001 ***	7.547 <0.001 ***	6320.867 <0.001 ***	5.658 <0.001 ***	2.564 <0.01 **
Cd Plant (underground)	1509.490 <0.001 ***	46.931 <0.001 ***	71,657.740 <0.001 ***	3.132 <0.01 **	9897.477 <0.001 ***	116.628 <0.001 ***	3.184 <0.001 ***
Transport factor	1478.382 <0.001 ***	7.169 <0.001 ***	25,960.472 <0.001 ***	7.820 <0.001 ***	6539.869 <0.001 ***	5.611 <0.001 ***	7.179 <0.001 ***

Note: The first line is the *F* value, and the second line is the *p*-value. ‘*’ represents significance. * *p* < 0.05, ** *p* < 0.01, *** *p* < 0.001.

**Table 3:** The second stage variance analysis table.

Index	Plant	Biochar	AMF	Plant × Biochar	Plant × AMF	AMF × Biochar	Plant × Biochar × AMF
Dry weight (aboveground)	52.303 <0.001 ***	54.383 <0.001 ***	73.934 <0.001 ***	2.371 *<0.077*	21.024 <0.001 ***	1.018 0.389	0.440 0.725
Dry weight (underground)	1.157 *0.285*	50.525 <0.001 ***	104.858 <0.001 ***	2.486 *<0.067*	31.521 <0.001 ***	0.742 0.530	0.340 *0.796*
Cd Plant (aboveground)	2107.398 <0.001 ***	65.384 <0.001 ***	8.832 <0.001 ***	0.08 *0.971*	6320.867 <0.001 ***	0.365 *0.778*	0.048 *0.986*
Cd Plant (underground)	1247.556 <0.001 ***	71.860 <0.001 ***	13.292 <0.001 ***	5.498 0.002 **	9897.477 <0.001 ***	0.814 *0.490*	0.074 0.974
Transport factor	1.493 *0.225*	6.065 <0.001 ***	0.920 *0.340*	1.686 *0.177*	6539.869 <0.001 ***	0.416 *0.742*	0.057 *0.982*

Note: The first line is the *F* value, and the second line is the *p*-value. ‘*’ represents significance. ** *p* < 0.01, *** *p* < 0.001.

## Data Availability

For experimental data, please contact the corresponding author.

## Supplementary Materials

The following supporting information can be downloaded at: https://www.mdpi.com/article/10.3390/toxics11070582/s1, Supplementary File: Analysis of variance of test indexes.
