# Supplementary material for: Microorganisms and Biochar Improve the Remediation Efficiency of Paspalum vaginatum and Pennisetum alopecuroides on Cadmium-Contaminated Soil
Source: Toxics. 2023 Jul 4;11(7):582. doi: 10.3390/toxics11070582 (PMC10383370; doi:10.3390/toxics11070582)
Supplement: Supplementary file 1 [file toxics-11-00582-s001.zip › Supplementary File.pdf]

Supplementary File: Analysis of variance of test indexes

1. The first stage: Variance analysis table of each index of seashore paspalum and pennisetum alopecuroides.

Table S1. The first stage variance analysis table

Index	Seashore paspalum			Pennisetum alopecuroides		
	Microorganism	Cadmium	Micro + Cd	Microorganism	Cadmium	Micro + Cd
Cd (plant)	28.965	9118.062	13.907	18.296	12714.906	2.948
above-ground	<0.001***	<0.001***	<0.001***	<0.05*	<0.001***	<0.01**
Cd (plant)	19.917	65024.190	8.187	30.156	16480.676	11.629
underground	<0.001***	<0.001***	<0.001***	0.187	<0.001***	<0.001***
Dry weight	44.971	17.746	0.387	57.311	315.254	6.331
above-ground	<0.001***	<0.001***	0.972	<0.001***	<0.001***	<0.001***
Dry weight	9.456	58.219	1.492	87.350	50.375	11.683
underground	<0.001***	<0.001***	0.151	<0.001***	<0.001***	<0.001***
Transport	9.222	23874.142	6.940	4.819	4445.239	5.552
factor	<0.001***	<0.001***	<0.001***	<0.05*	<0.001***	<0.001***

Note:The first row represents *F* value, and the second row represents *P* value. '*' represents significance.

2. The second stage: Variance analysis table of each index of seashore paspalum and pennisetum alopecuroides.

Table S2.The second stage variance analysis table

Index	Seashore paspalum			Pennisetum alopecuroides		
	Biochar	AMF	Biochar + AMF	Biochar	AMF	Biochar + AMF
Cd (plant)	43.507	5.639	0.218	25.463	3.709	0.194
above-ground	<0.001***	<0.001***	0.884	<0.001***	<0.01**	0.900
Cd (plant)	97.183	39.053	1.134	43.985	7.228	0.502
underground	<0.001***	<0.001***	0.343	<0.001***	<0.001***	0.682
Dry weight	35.403	6.254	1.283	50.003	11.473	0.184
above-ground	<0.001***	<0.001***	0.289	<0.001***	<0.001***	0.907
Dry weight	41.240	12.780	0.598	29.687	6.103	1.034
underground	<0.001***	<0.001***	0.619	<0.001***	<0.001***	0.384
Transport	5.670	0.664	0.581	5.064	0.660	0.124
factor	<0.01**	0.721	0.630	<0.01**	0.724	0.945

Note:The first row represents *F* value, and the second row represents *P* value. '*' represents significance.
